# Inflatable Metamorphic Origami

**DOI:** 10.34133/research.0133

**Published:** 2023-05-04

**Authors:** Sen Wang, Peng Yan, Hailin Huang, Ning Zhang, Bing Li

**Affiliations:** ^1^ School of Mechanical Engineering and Automation, Harbin Institute of Technology, Shenzhen 518052, P.R. China.; ^2^ Guangdong Provincial Key Laboratory of Intelligent Morphing Mechanisms and Adaptive Robotics, Harbin Institute of Technology, Shenzhen 518052, P. R. China.; ^3^ State Key Laboratory of Robotics and System, Harbin Institute of Technology, Harbin 150001, P.R. China.

## Abstract

This study created a new type of inflatable metamorphic origami that has the advantage of being a highly simplified deployable system capable of realizing multiple sequential motion patterns with a monolithic actuation. The main body of the proposed metamorphic origami unit was designed as a soft inflatable metamorphic origami chamber with multiple sets of contiguous/collinear creases. In response to pneumatic pressure, the metamorphic motions are characterized by an initial unfolding around the first set of contiguous/collinear creases followed by another unfolding around the second set of contiguous/collinear creases. Furthermore, the effectiveness of the proposed approach was verified by constructing a radial deployable metamorphic origami for supporting the deployable planar solar array, a circumferential deployable metamorphic origami for supporting the deployable curved-surface antenna, a multi-fingered deployable metamorphic origami grasper for grasping large-sized objects, and a leaf-shaped deployable metamorphic origami grasper for capturing heavy objects. The proposed novel metamorphic origami is expected to serve as a foundation for designing lightweight, high-deploy/fold-ratio, low-energy-consumption space deployable systems.

## Introduction

Deployable systems are thought to be an ideal solution for developing special intelligent systems that must change shape to a smaller size for easy storage and transportation while being deployed into a larger volume configuration to reach a large working space [1–4]. Deployable space systems [5–8], soft morphing robots [9–12], origami-inspired robots [13–17] and structures [18–22], and metamaterials [23–27] are some typical examples. The ability to deploy these systems makes them extremely flexible and adaptable to complex working environments.

**Fig. 1.**
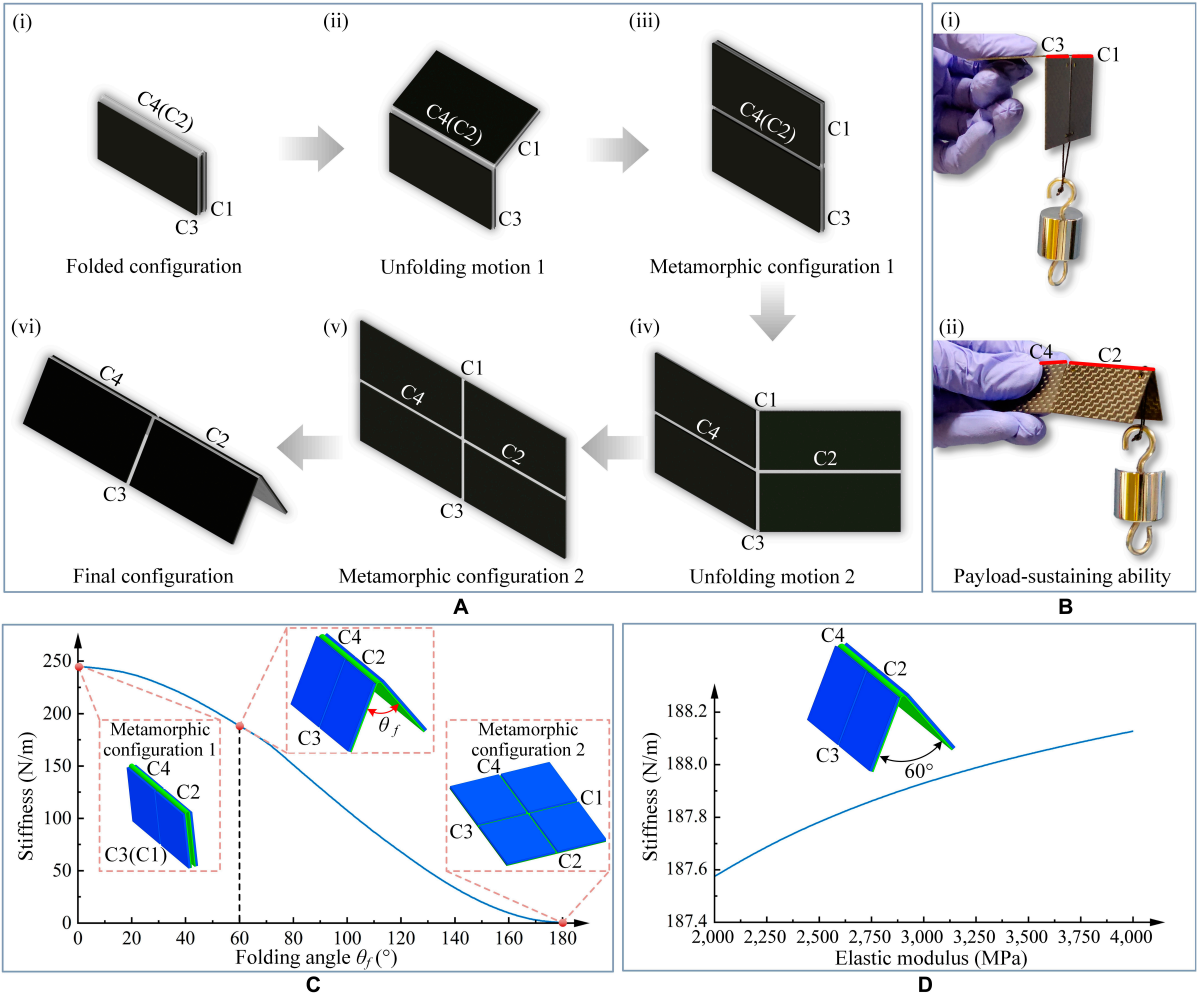
The metamorphic origami unit. (A) The metamorphic unfolding process. (B) The payload-sustaining ability. (C) The stiffness behavior of the metamorphic origami unit at different folding angles. (D) The stiffness behavior of the metamorphic origami unit at different elastic modulus values.

Traditionally, deployable systems were always designed with fixed mobility [28–30]. Such fixed-mobility deployable systems are capable of realizing only a unique motion pattern, which can only conduct a single deploying or folding motion [31,32]. However, many systems may require additional manipulation motion tasks to be performed after the deployment motion. Therefore, traditionally, such deployable systems were mostly designed with an independent deployment motion and a manipulation motion, both of which were actuated by two separate actuation systems [33]. Such a design, however, had a greater energy consumption, higher complexity, and increased massiveness. In order to realize changeable mobility for a given mechanism, the metamorphic mechanism was first proposed by Dai and Jones [34], and many metamorphic systems have been developed in recent years [35,36]. However, a metamorphic system always needs an additional actuation system to adjust its mobility, such that the system is still very complicated [37]. In this context, a metamorphic system capable of generating multiple sequential motion patterns under a monolithic actuation source could be a suitable solution, although this research area remains relatively unexplored. A simple metamorphic origami unit in Fig. 1 is a typical example of such a system, its mobility multi-furcation property could inspire a set of novel deployable systems with multiple sequential motion patterns.

The inflatable metamorphic origami proposed in the present study is capable of generating sequential deploying and bending motions in response to pneumatic pressure. This special pattern of motion is characterized by a sequence of motions comprising an initial unfolding around the first set of contiguous/collinear creases followed by another unfolding around another set of contiguous/collinear creases as the pneumatic pressure increases. Next, to verify the effectiveness of the proposed novel metamorphic origami, the following were developed: a radial deployable metamorphic origami for supporting the deployable planar solar array, a circumferential deployable metamorphic origami for supporting the deployable curved-surface antenna, a multi-fingered deployable metamorphic origami grasper for grasping large-sized objects, and a leaf-shaped deployable metamorphic origami grasper for capturing heavy objects. All these deployable systems demonstrated that each metamorphic origami could, under a single actuation, realize multiple sequential motion patterns such as the deployment motion, bending motion for grasping objects, or multiple stable configurations for supporting loads.

## Results

### Origami with mobility multi-furcation

Figure 1 shows a metamorphic origami unit with mobility multi-furcation. The origami unit comprised 4 facets and 4 creases. In the folded configuration [(i) in Fig. 1A], the unit could be unfolded around the contiguous creases C2 and C4; in this configuration, creases C1 and C3 are neither contiguous nor collinear. When the unit begins to unfold [(ii) in Fig. 1A], it would, at a certain point, reach a unique intermediate configuration in which creases C1 and C3 are collinear [(iii) in Fig. 1A]. This configuration is the first metamorphic configuration of the origami unit. In this metamorphic configuration, the origami has a mobility bifurcation, such that the origami could continue to unfold around the contiguous creases C2 and C4 or unfold around the new collinear creases C1 and C3. If the origami was unfolded around the collinear creases C1 and C3, then the creases C2 and C4 would no longer be contiguous, thereby preventing the origami unit from being rotated around creases C2 and C4 again [(iv) in Fig. 1A]. The origami is incapable of sustaining load as it is possible to rotate it around creases C1 and C3 [(i) in Fig. 1B]. The unit then continues to unfold to another special intermediate configuration in which creases C2 and C4 are also collinear. This configuration is the second metamorphic configuration of the origami [(v) in Fig. 1A]. In the metamorphic configuration 2, the origami also has a mobility bifurcation. When the origami is rotated around the collinear creases C2 and C4, it moves into another motion pattern until the creases C1 and C3 are no longer collinear [(vi) in Fig. 1A]. After that, the origami would not rotate around creases C1 and C3 again, i.e., the rotation around creases C1 and C3 would be locked. In other words, origami has load-bearing ability in this configuration [(ii) in Fig. 1B and Movie S1]. The metamorphic origami unit with 4 facets and 4 creases was evaluated quantitatively using the ABAQUS software. The simulation process is illustrated in Note S2. Figure 1C illustrates the stiffness of the metamorphic origami unit when the origami unit was folded around the collinear creases C2 and C4 from the metamorphic configuration. The stiffness was observed to increase as the folding angle was decreased. This property demonstrates that, in the metamorphic configuration, the stiffness is close to zero, which enables the origami unit to be folded around the collinear creases C1 and C3. If the origami unit is folded around another set of collinear creases C2 and C4, the creases C1 and C3 no longer remain collinear, and the stiffness is increased further. This implies that the origami unit would no longer be folded around creases C1 and C3 again, i.e., the origami unit has force sustainability. In addition, it was demonstrated that, as the elastic modulus of the origami unit's facet panels increased, the origami unit's stiffness also increased (Fig. 1D). Therefore, a facet with an adequate modulus of elasticity had to be designed if large stiffness was required.

**Fig. 2.**
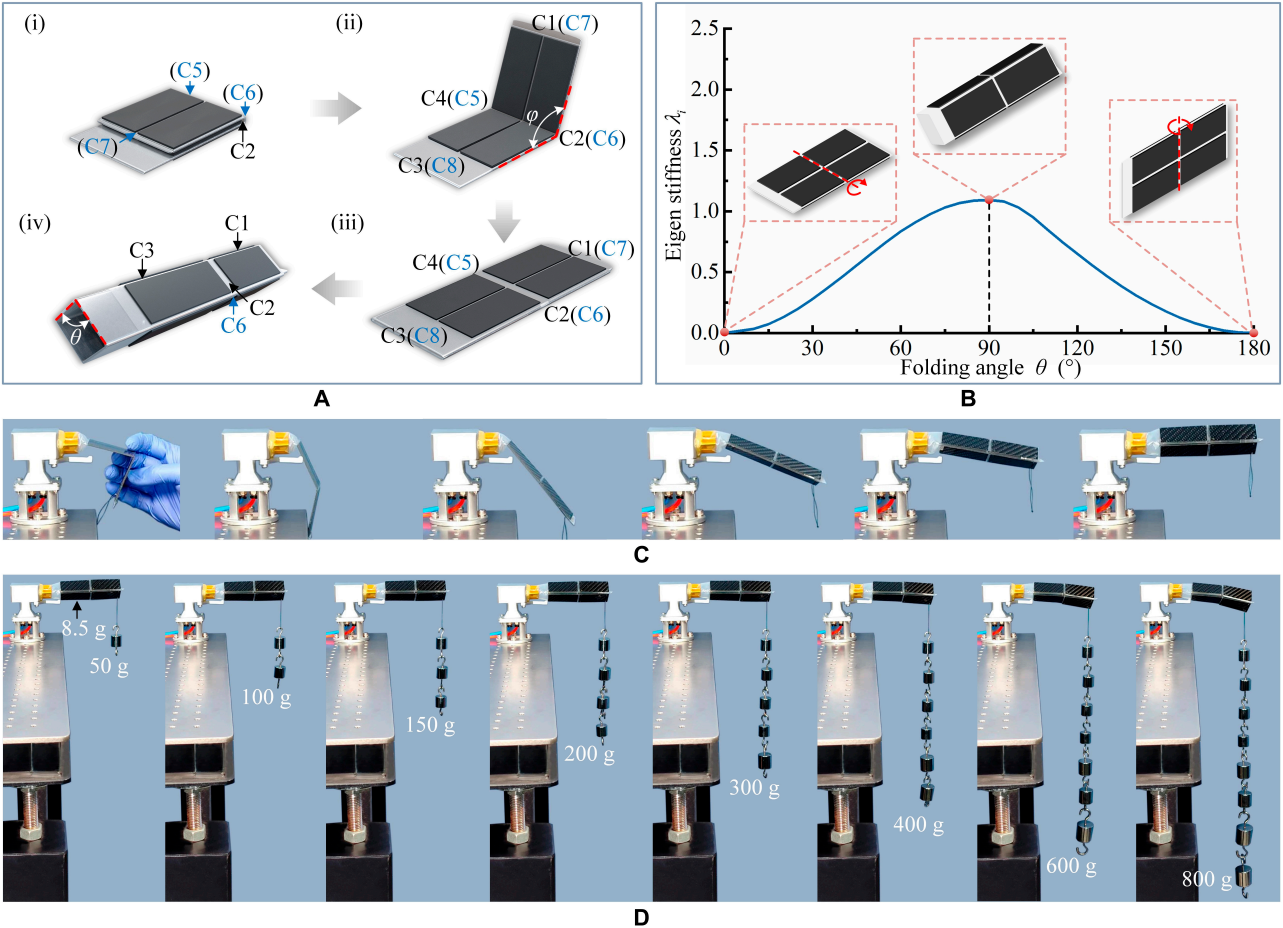
The stiffness behavior of an inflatable metamorphic origami unit. (A) The deployment process of an inflatable metamorphic origami unit. (B) The stiffness behavior of the inflatable metamorphic origami unit. (C) The deployment process of an inflatable metamorphic origami prototype unit. (D) The load capacity of the inflatable metamorphic origami.

The inflatable metamorphic unit was designed based on the metamorphic origami unit with 4 facets, and a soft pneumatic chamber was used to drive the inflatable origami unit to move from one movement pattern to another. Figure 2A depicts an inflatable metamorphic origami unit with 8 identity rectangular facets attached to a polyethylene (PE) lay flat tube. The soft chamber of the origami was driven by a monolithic pneumatic actuator. Because the air pressure in the chamber was monotonic increasing, then the air capacity of the chamber is also a monotonic increasing process. The inflatable metamorphic origami is initially folded with 2 sets of contiguous creases [(i) in Fig. 2A], namely, the collinear creases C5 and C6, which are contiguous with the collinear creases C2 and C4, such that the inflatable origami can only be unfolded around these 2 sets of contiguous creases in response to increasing pneumatic pressure [refer to (ii) in Fig. 2A]. The folding angle *θ* is large in this stage, and the extension angle *φ* is small; based on the stiffness behavior described in Note S2, the stiffness along the extension motion is weaker than the stiffness along the inflation motion of the chamber; thus, the motion in this stage is extension-dominant. When the inflatable metamorphic origami unit reaches the metamorphic configuration in which the creases C1 (C7) and C3 (C8) are collinear [(iii) in Fig. 2A], the origami will change the motion pattern of unfolding around the creases C2 and C4 to that around the creases C1 (C7) and C3 (C8), e.g., the motion pattern changes from extension-dominant to inflation-dominant. When the air input reaches the maximum capacity of the chamber, the origami stops inflating. The origami chamber inflates to maximum capacity when the rhomboid cross-section of the chamber reaches the maximum area value, e.g., folding angle *θ* = 90° [(iv) in Fig. 2A]. The equivalent pin-jointed truss frame model was established to show the stiffness behavior of this inflatable metamorphic origami unit during the metamorphic deployment process, which verifies that when this inflatable metamorphic origami unit is at maximum capacity state, i.e., folding angle *θ* = 90°, it has maximum stiffness (Fig. 2B and Note S3). When the inflatable metamorphic origami unit is at minimum capacity, i.e., the folding angle *θ* = 0° or *θ* = 180°, the inflatable metamorphic origami is at 2 metamorphic positions, the stiffness of the inflatable metamorphic origami unit in these 2 states are approximate to 0, and the unit can be rotated around the contiguous creases. A physical prototype was also fabricated to demonstrate the sequential deployment process of the inflatable metamorphic origami unit (Fig. 2C) and the load capacity in the deployed configuration (Fig. 2D). The weight of the unit was 8.5 g. Initially, the unit could be folded around the contiguous creases with no payload-sustaining ability. When the unit was inflated, it could sustain a payload of approximately 400 g, which is around 47 times its weight, without much deformation. When the payload continuously increased beyond this weight, the unit generated evident deformation (800 g) (Movie S2).

### Radial deployable metamorphic origami

**Fig. 3.**
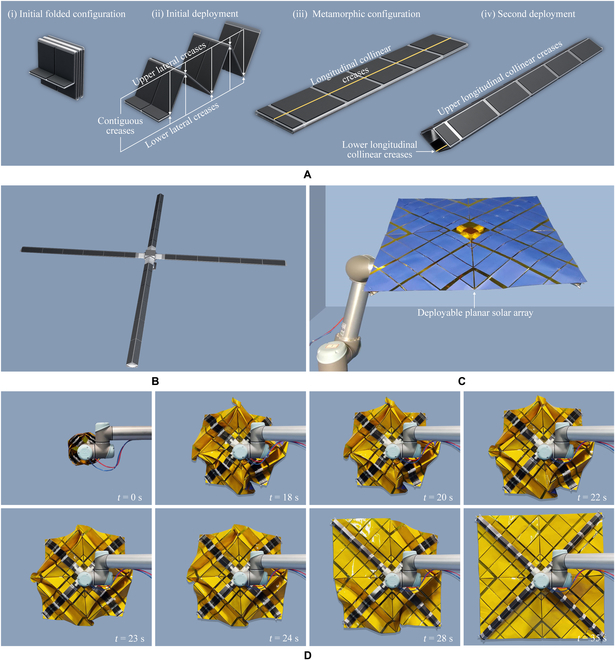
Radial deployable metamorphic origami for supporting a large deployable planar solar array. (A) The deployable branch of the developed radial deployable metamorphic origami. (B) The radial deployable metamorphic origami with 4 branches. (C) The experimental system of the developed radial deployable metamorphic origami for supporting a deployable solar array. (D) The deployment process of the physical prototype.

The inflatable metamorphic origami developed in the present study is capable of realizing an initial deployment motion followed by a stable load-bearing status and could, therefore, be used for supporting large-scale deployable solar arrays in space applications (Movie S3). A branch unit of the developed metamorphic origami is depicted in Fig. 3A. The branch unit of the metamorphic origami was designed as a soft origami chamber with a rhombic cross-section in the deployed configuration, and all creases in this origami are rigid creases (Note S1). Initially, the branch unit is folded into a compact zigzag-shaped configuration [(i) in Fig. 3A]. In this configuration, the upper lateral creases and the lower lateral creases at the 2 ends of each rigid facet are contiguous, and it is possible to deploy the branch unit around these contiguous creases to generate the first deployment motion in response to the air pressure [(ii) in Fig. 3A]. After the origami continues to deploy into the metamorphic configuration with longitudinal collinear creases [(iii) in Fig. 3A], the upper lateral creases and the lower lateral creases are no longer contiguous as the air pressure continues to increase, causing the rotations around the first set of contiguous creases to be locked [(iv) in Fig. 3A]. Using this branch, the radial deployable metamorphic origami with 4 branch units was developed to support a large deployable planar solar array (Fig. 3B and C). The deployment process of this deployable solar array using the developed metamorphic origami is illustrated in Fig. 3D. This origami reaches a considerably high deploy/fold ratio; the deploy/fold ratio for each branch was approximately 38.8, while the deploy area/fold area ratio for the developed radial deployable metamorphic origami with 4 branches supporting a deployable planar solar array was approximately 20.8.

The metamorphic origami developed in this study can generate sequential unfolding motions around various sets of contiguous/collinear creases, which were characterized by a large initial deployment motion followed by a stable load-bearing status. The creases in this origami could only generate rotational motions for folding/unfolding. Furthermore, due to the flexibility of the soft creases, this origami had a slight thickness accommodation, which is especially important for the folding/unfolding motion of contiguous creases. However, the creases must be precise to maintain load-bearing capability and provide strict geometric constraints to the origami to generate a kinematically guided origami folding, similar to a rigid mechanism. Therefore, this type of origami crease was termed a rigid crease (Note S1).

### Circumferential deployable metamorphic origami

**Fig. 4.**
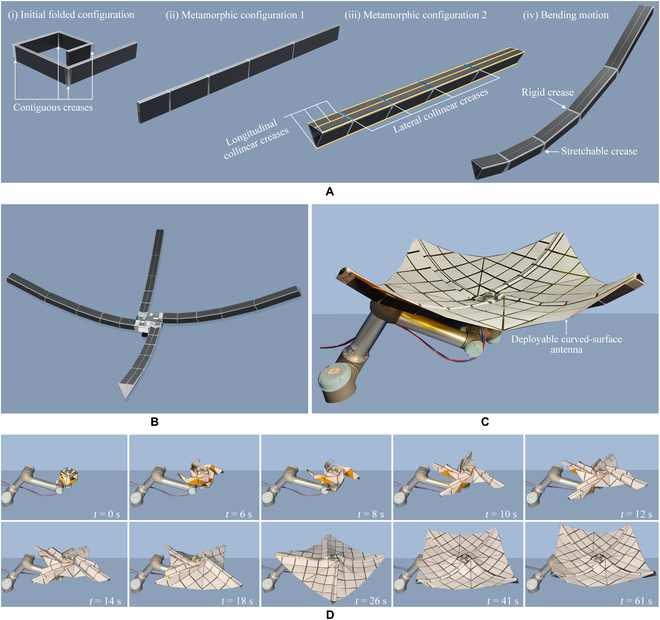
The circumferential deployable metamorphic origami for supporting a deployable curved-surface antenna. (A) The deployable branch of the developed circumferential deployable metamorphic origami. (B) The circumferential deployable metamorphic origami with 4 branches. (C) The experimental system of the developed circumferential deployable metamorphic origami. (D) The deployment process of the physical prototype.

In order to demonstrate the versatility of the developed metamorphic origami, a circumferential deployable metamorphic origami was developed to support a large deployable curved-surface antenna, which would be able to generate an initial deployment motion of a large magnitude followed by a bending motion for adjusting the curvature of the supporting deployable curved-surface antenna (Movie S4). Similar to radial deployable metamorphic origami, circumferential deployable metamorphic origami also comprises 4 branches. In the initial folded configuration, these branches are folded around the rigid folding contiguous creases that surround a square mounting base [Fig. 4A(i) and Fig. S6H]. In response to increasing air pressure, the branch first deploys into an unbent status [(ii) in Fig. 4A], which is a metamorphic configuration with 4 longitudinal collinear creases. Next, the branch continues to inflate around the 4 longitudinal collinear creases in response to the air pressure until it reaches the second metamorphic configuration in which the cross-section of the branch becomes triangular [(iii) in Fig. 4A]. In this configuration, the creases for the initial deployment are no longer contiguous, causing the unfolding around these creases to be locked. In the second metamorphic configuration, the origami reaches new lateral collinear creases, enabling the branch to be unfolded around the lateral collinear creases as the air pressure increases. In order to realize the bending motion around the lateral collinear creases, the design used stretchable creases, capable of generating both folding/unfolding motion and stretching motion owing to their wrinkled structure. Different from what was reported in a previous study [13], this stretching motion realized in the present study was not due to the stretching of the soft body and rather due to the stretching motion of the wrinkled structure (Note S1). In the first deployment motion, this metamorphic origami branch was deployed around the rigid creases, similar to a rigid mechanism. The stretchable creases provided adaptive accommodation motion without introducing kinematic constraints, and this was termed a rigid-dominant motion. When the branch was moved to the metamorphic configuration 2 [(iii) in Fig. 4A], it could be unfolded around the lateral collinear creases to generate a bending motion. In this process, the lateral collinear creases worked as rigid folding creases, which ensured the kinematic constraints and precise motion. On the other hand, the stretchable creases worked in the stretching motion to provide the bending actuation force. The bending motion of the origami branch was, therefore, a superimposition of the rigid geometry-driven motion generated by rigid creases and the stretching motion generated from the stretchable creases [(iv) in Fig. 4A and Note S1]. The bending motion of this origami was, therefore, termed the rigid-flexible coupling motion. This branch was used for developing a circumferential deployable metamorphic origami with 4 branch units to support a large deployable curved-surface antenna (Fig. 4B and C). The deployment process of the deployable curved-surface antenna driven by this metamorphic origami is illustrated in Fig. 4D. The circumferential deployable metamorphic origami reached a considerably high deploy/fold ratio of 29.1 for the prototype developed in the present study.

### Multi-fingered deployable metamorphic origami grasper

**Fig. 5.**
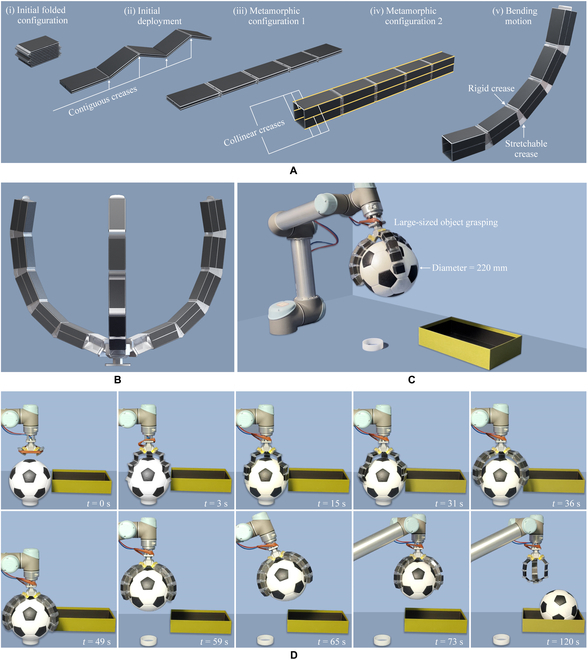
The multi-fingered deployable metamorphic origami grasper. (A) A branch of the multi-fingered deployable metamorphic origami grasper. (B) The metamorphic origami grasper with 3 branches. (C) The experimental system of the multi-fingered deployable metamorphic origami grasper. (D) The process of grasping exhibited by the physical prototype.

The developed circumferential deployable metamorphic origami exhibited sequential motion patterns under a single pneumatic actuation that could be applied to soft deployable robotic systems. Two types of deployable metamorphic origami graspers were developed in the present study to demonstrate the potential versatile applications. The first type of grasper was a multi-fingered deployable grasper. The architecture of this multi-fingered metamorphic origami grasper was similar to that of the circumferential deployable metamorphic origami, which could generate sequential deployment motions to realize a large working space motion and a bending motion for grasping large-sized objects. Initially, the branch is folded into a zigzag-shaped configuration [(i) in Fig. 5A]. As the air pressure increased, the branch was first deployed into an unbend configuration around the contiguous creases (rigid-dominant), which was followed by a motion in which the branch reached the first unbend metamorphic configuration with 6 longitudinal collinear creases [(iii) in Fig. 5A]. Subsequently, the origami continued to inflate to the second metamorphic configuration with a rectangular cross-section by unfolding around these longitudinal collinear creases [(iv) in Fig. 5A]; this process was also termed a rigid-dominant process. In the second metamorphic configuration, the creases for the initial deployment [(ii) in Fig. 5A] no longer remained contiguous, causing the unfolding around these creases to be locked. With the continuous increase in the air pressure, the stretchable creases begin working, thereby generating a bending motion for grasping large -sized objects [(v) in Fig. 5A]. This bending motion process is a rigid-flexible coupling motion process generated by both rigid creases and stretchable creases. This branch was then used for developing a triple-fingered deployable grasper for grasping large -sized objects (Fig. 5B and C). Next, to demonstrate the size adaptability of this metamorphic origami grasper in grasping large -sized objects, grasping experiments were conducted (Movie S5). The entire grasping process of this grasper based on metamorphic origami is illustrated in Fig. 5D. The grasper was observed to be capable of grasping objects 3 times its size.

### Leaf-shaped deployable metamorphic origami grasper

**Fig. 6.**
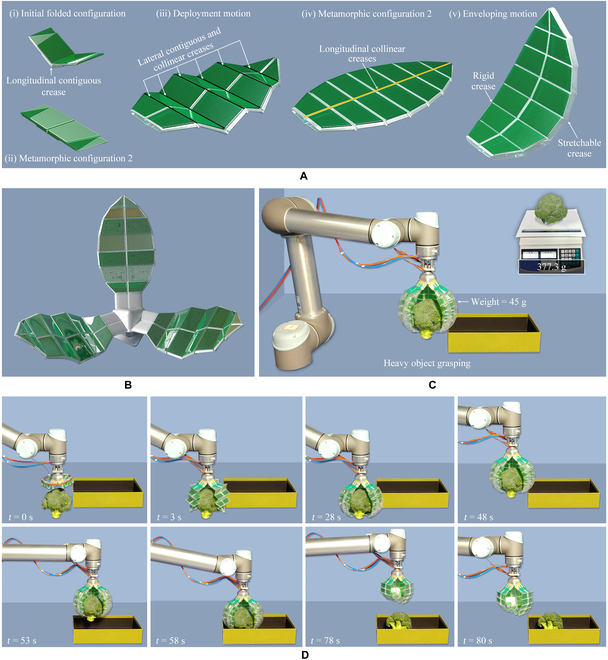
The leaf-shaped deployable metamorphic origami grasper. (A) A branch of the developed leaf-shaped deployable metamorphic origami grasper. (B) The leaf-shaped deployable metamorphic origami grasper with 3 branches. (C) The experimental system of the developed leaf-shaped deployable metamorphic origami grasper. (D) The process of grasping exhibited by the physical prototype.

The second type of grasper was the leaf-shaped deployable grasper, which had branches with a vegetal leaf-shaped structure. The grasper comprised 3 leaf-shaped branches, which could generate an enveloping motion for grasping heavy objects (Fig. 6B). Initially, the branches are folded around a mounting base [(i) in Fig. 6A and Fig. S8G]. The branches could then be deployed around the longitudinal contiguous creases circumferentially into the first metamorphic configuration [(i) and (ii) in Fig. 6A]. Afterward, the branches could be continued to be deployed around another set of lateral contiguous and collinear creases [(iii) in Fig. 6A] to the second metamorphic configuration [(iv) in Fig. 6A]. In the second metamorphic configuration, a new longitudinal collinear crease was reached, which enabled the generation of a slight folding motion of the origami around this collinear crease; combined with bending motion, these 2 motions achieve envelope grasping together [(v) in Fig. 6A]. This branch was then used for developing a deployable grasper for grasping heavy objects (Fig. 6B to D). The diameter of the grasper in folded configuration was 8 cm. The diameter of the object was approximately 16 cm, which is 2 times that of the grasper in the folded configuration. The total weight of the 3 branches was 45 g, while the grasper could grasp an object weighing up to 377.3 g, which is 8.3 times the weight of the grasper (Movie S6).

## Conclusion

The current study created a new type of inflatable metamorphic origami that could achieve sequential functionally different motion patterns with a single actuator. The developed metamorphic origami unit has applications such as the basic module of high-deploy/fold-ratio, lightweight, and large-workspace deployable robotic systems that require performing both deployment and other manipulation motions. The present study also demonstrated a few robotic applications of the developed pneumatic-driven metamorphic origami unit. The versatility of the metamorphic origami systems was demonstrated by constructing a set of deployable robotic systems based on the metamorphic origami units, namely, a radial deployable metamorphic origami for supporting a deployable planar solar array, a circumferential deployable metamorphic origami for supporting a deployable curved-surface antenna, a multi-fingered deployable metamorphic origami grasper for grasping large-sized objects, and a leaf-shaped deployable metamorphic origami grasper for capturing heavy objects. All these robots demonstrated that each of the developed metamorphic origami units could realize multiple sequential motion patterns, such as the deployment motion to reach a large working space, a bending motion for grasping objects, or multiple stable configurations for supporting loads under a single actuation. The design approach proposed in the present study is also potentially applicable to other existing origami structures. The proposed approach offers a novel solution for designing lightweight deployable systems capable of being deployed into a considerably large-volume working configuration. This would be particularly advantageous for application in space robots, in which the storage cabin space of the rocket is quite small while the operation is in a large-volume status when in orbit. Future work could include improving self-folding ability, developing novel durable materials for soft chamber fabrication, and developing lightweight, high-stiffness rigid panels for origami facets.

## Materials and Methods

### Materials for the developed deployable metamorphic origami

The flexible PE lay flat tubes (thickness: 0.1 mm) were used for fabricating the pneumatic inflatable soft origami chambers. The carbon fiber sheets (thickness: 0.4 mm for the radial and circumferential deployable metamorphic origami and 0.3 mm for the multi-fingered deployable metamorphic origami grasper) and the flexible polyvinyl chloride sheets (thickness: 0.5 mm for the leaf-shaped deployable metamorphic origami) were used for fabricating the rigid facets of the metamorphic origami. The mounting bases of the metamorphic origami were fabricated using high-toughness photosensitive resin through 3D printing technology.

### Fabrication of the developed deployable metamorphic origami

The rigid facets were attached to the flexible PE lay flat tubes to obtain the creases of the metamorphic origami between the adjacent facets. The metamorphic origami was fabricated according to the following rules:

1. Kapton double-sided sticky tapes (0.1 mm thick) were used to attach the rigid facets to the inflatable soft PE lay flat tubes (Fig. S4A).

2. The terminal of the PE lay flat tube was sealed using a heat sealer (PFS-300; Baijie Ltd.) to form the pneumatic chambers for each branch (Fig. S4B).

3. In order to realize the metamorphic origami with both deployment motion and bending motion, the stretchable creases were first squeezed to form wrinkles with the same gap as that in the rigid creases. The rigid creases responsible for the geometric folding/unfolding were glued using the 3M610–1PK tape (Scotch; 3M Ltd.) to ensure their non-stretchability; consequently, these rigid creases could generate pure folding/unfolding motion (Fig. S4C). The stretchable creases owed their stretchability and foldability to the wrinkled structure, which provided space for the bending motion. The bending motion could then be generated through the folding/unfolding motion of the rigid creases combined with the stretching motion of the stretchable creases.

4. In order to obtain the rigid creases of the radial deployable metamorphic origami, the outer surface of the facets was glued using the 3M610–1PK tape (Scotch; 3M Ltd.) to render it non-stretchable (Fig. S4C). This also improved the stiffness of the origami.

5. The non-sealed ends of the origami branches were glued on the mounting interfaces of the base using double-sided sticky tape (Fig. S4D).

6. Sil-Poxy silicone adhesive (Smooth-On Inc.) was also used to seal the mounting interfaces of all branches to produce a deployable metamorphic origami with adequate airtightness (Fig. S4E).

### Actuation for the deployable metamorphic origami

All deployable metamorphic origami designed in the present study were controlled using a pneumatic source system. Each deployable metamorphic origami was actuated using a single pneumatic source through a soft polyurethane tube. The air pressure was controlled using a servo valve.

### Stiffness analysis

The stiffness simulations were performed using the commercially available FEA software named ABAQUS (Dassault System). Rectangular rigid panels were attached to a soft membrane to establish the origami simulation model. The rigid facets were formed using an acrylic plate material, while the membrane was formed of paper material. The elastic modulus values of the rigid facets and the soft membrane were set to 3,300 MPa and 0.5 MPa, respectively, while the Poisson ratio for both rigid facets and the soft membrane was set to 0.2. Consequently, the crease could be obtained between the 2 adjacent rigid panels due to the difference between the stiffness of the rigid panels and that of the membrane (Fig. S2B and Note S2). In order to perform the stiffness simulation, the 2 edges of the facets of the metamorphic origami unit were fixed, and 2 identical loading forces were applied to the 2 edges of the other 2 facets opposite to the fixed ends. The loads were applied along the direction of the bisector of the folding angle (Fig. S2D). The stiffness of a point was calculated by measuring the displacement of the force loading point along the direction of the loads (Note S2). The stiffness behavior of the inflatable metamorphic origami unit was analyzed by establishing the equivalent pin-jointed truss frame model (Note S3).

## Data Availability

All data needed to evaluate the conclusions in the paper are present in the paper and/or the Supplementary Materials. Additional data related to this paper may be requested from the authors.

## Supplementary Materials

Supplementary Materials Note S1. Design of the origami creases. Note S2. Simulations of the origami cell, metamorphic origami unit, and inflatable metamorphic origami unit. Note S3. Stiffness analysis of the inflatable metamorphic origami unit. Note S4. Fabrication of the radial deployable metamorphic origami. Note S5. Fabrication of the circumferential deployable metamorphic origami. Note S6. Fabrication of the multi-fingered deployable metamorphic origami grasper. Note S7. Fabrication of the leaf-shaped deployable metamorphic origami grasper. Note S8. The self-folding ability design of the metamorphic origami. Fig. S1. Design of the origami creases. Fig. S2. Simulations of the origami cell and metamorphic origami unit. Fig. S3. Geometric model of the inflatable metamorphic origami unit. Fig. S4. The fabrication process of the deployable metamorphic origami prototype. Fig. S5. The fabrication process of the radial deployable metamorphic origami. Fig. S6. The fabrication process of the circumferential deployable metamorphic origami. Fig. S7. The fabrication process of the multi-fingered deployable metamorphic origami grasper. Fig. S8. The fabrication process of the leaf-shaped deployable metamorphic origami grasper. Fig. S9. The arrangement of the elastic steel wires for self-folding. Movie S1. Stiffness property of a metamorphic origami unit. Movie S2. Stiffness property of an inflatable metamorphic origami unit. Movie S3. Deployment process of a radial deployable metamorphic origami. Movie S4. Deployment process of a circumferential deployable metamorphic origami. Movie S5. Multi-fingered deployable metamorphic origami grasper. Movie S6. Leaf-shaped deployable metamorphic origami grasper.
