# Supplementary material for: Inflatable Metamorphic Origami
Source: Research (Wash D C). 2023 May 4;6:0133. doi: 10.34133/research.0133 (PMC10204744; doi:10.34133/research.0133)
Supplement: Supplementary Materials — Note S1. Design of the origami creases. Note S2. Simulations of the origami cell, metamorphic origami unit, and inflatable metamorphic origami unit. Note S3. Stiffness analysis of the inflatable metamorphic origami unit. Note S4. Fabrication of the radial deployable metamorphic origami. Note S5. Fabrication of the circumferential deployable metamorphic origami. Note S6. Fabrication of the multi-fingered deployable metamorphic origami grasper. Note S7. Fabrication of the leaf-shaped deployable metamorphic origami grasper. Note S8. The self-folding ability design of the metamorphic origami. Fig. S1. Design of the origami creases. Fig. S2. Simulations of the origami cell and metamorphic origami unit. Fig. S3. Geometric model of the inflatable metamorphic origami unit. Fig. S4. The fabrication process of the deployable metamorphic origami prototype. Fig. S5. The fabrication process of the radial deployable metamorphic origami. Fig. S6. The fabrication process of the circumferential deployable metamorphic origami. Fig. S7. The fabrication process of the multi-fingered deployable metamorphic origami grasper. Fig. S8. The fabrication process of the leaf-shaped deployable metamorphic origami grasper. Fig. S9. The arrangement of the elastic steel wires for self-folding. Movie S1. Stiffness property of a metamorphic origami unit. Movie S2. Stiffness property of an inflatable metamorphic origami unit. Movie S3. Deployment process of a radial deployable metamorphic origami. Movie S4. Deployment process of a circumferential deployable metamorphic origami. Movie S5. Multi-fingered deployable metamorphic origami grasper. Movie S6. Leaf-shaped deployable metamorphic origami grasper. [file research.0133.f1.zip › Supplementary Materials.docx]

Supplementary Materials for

Inflatable metamorphic origami

Sen Wang1†, Peng Yan1†, Hailin Huang1,2*, Ning Zhang1, Bing Li,1,2,3

1School of Mechanical Engineering and Automation, Harbin Institute of Technology, Shenzhen, 518052, P.R. China.

2Guangdong Provincial Key Laboratory of Intelligent Morphing Mechanisms and Adaptive Robotics, Harbin Institute of Technology, Shenzhen, 518052, P. R. China.

3State Key Laboratory of Robotics and System, Harbin Institute of Technology, Harbin 150001, P.R. China.

*Correspondence should be addressed to Hailin Huang; huanghailin@hit.edu.cn

†These authors contributed equally to this work

The PDF file includes:

Note S1: Design of the origami creases.

Note S2: Simulations of the origami cell, metamorphic origami unit, and inflatable metamorphic origami unit.

Note S3: Stiffness analysis of the inflatable metamorphic origami unit.

Note S4: Fabrication of the radial deployable metamorphic origami.

Note S5: Fabrication of the circumferential deployable metamorphic origami.

Note S6: Fabrication of the multi-fingered deployable metamorphic origami grasper.

Note S7: Fabrication of the leaf-shaped deployable metamorphic origami grasper.

Note S8: The self-folding ability design of the metamorphic origami.

Fig. S1: Design of the origami creases.

Fig. S2: Simulations of the origami cell and metamorphic origami unit.

Fig. S3: Geometric model of the inflatable metamorphic origami unit.

Fig. S4: The fabrication process of the deployable metamorphic origami prototype.

Fig. S5: The fabrication process of the radial deployable metamorphic origami.

Fig. S6: The fabrication process of the circumferential deployable metamorphic origami.

Fig. S7: The fabrication process of the multi-fingered deployable metamorphic origami grasper.

Fig. S8: The fabrication process of the leaf-shaped deployable metamorphic origami grasper.

Fig. S9: The arrangement of the elastic steel wires for self-folding.

Other Supplementary Material for this manuscript includes the following:

Movie S1: Stiffness property of a metamorphic origami unit.

Movie S2: Stiffness property of an inflatable metamorphic origami unit.

Movie S3: Deployment process of a radial deployable metamorphic origami.

Movie S4: Deployment process of a circumferential deployable metamorphic origami.

Movie S5: Multi-fingered deployable metamorphic origami grasper.

Movie S6: Leaf-shaped deployable metamorphic origami grasper.

Note S1: Design of the origami creases

The origami prototype described in the present study was obtained by attaching the rigid facets to a soft membrane. Accordingly, the creases were represented by the gaps between the adjacent rigid facets. The folding/unfolding motion of a typical origami is generated by the rotational motion around the crease of two adjacent rigid facets and the slight deformation motion of the rigid facets. In the present study, however, the metamorphic origami was designed to have two types of creases. The first one was termed a rigid crease and was equivalent to the rotational motion of a rigid mechanism. This type of crease was responsible for generating a pure geometric folding/unfolding motion, as illustrated in Fig. S1A. The other type of crease was termed the stretchable crease and was designed to have a wrinkled structure that would enable both folding/unfolding motion and stretching motion, as illustrated in Fig. S1B. When the two types of creases are contiguous/collinear, the mobility intersection between the creases generates the geometric folding/unfolding motion dominated by the rigid crease. The origami could, therefore, be folded/unfolded synchronously around multiple creases if the creases were contiguous or collinear (refer to Fig. S1C).

Note S2: Simulations of the origami cell, metamorphic origami unit, and inflatable metamorphic origami unit

(1) Stiffness behavior simulation of the origami cell

Because of the difference in stiffness levels between the creases and facets, the folding/unfolding motion of the origami was generated by the rotation around the crease of the adjacent rigid facets. Fig. S2A shows an origami cell with one crease and two facets. To create the origami model, rectangular rigid facets were attached to a soft membrane. For this origami cell, the direction perpendicular to the plane of facet 2 has zero stiffness, while the direction that parallels the crease has the maximum stiffness. Thus, the stiffness of this origami cell is equal to the stiffness along the direction parallel to the crease . Suppose that one edge AB of facet 1 is fixed, and a loading force of angle () with respect to CD is applied to the center point of the edge CD of facet 2. Because the loading force can be divided into the normal component and the tangent component , facet 2 can be freely rotated around the crease with respect to facet 1 under the normal component , and elastic deformation of the facet is mainly generated by the tangent component . When angle , there only exists the elastic deformation for this origami cell.

In order to verify the folding pattern of the origami cell, the geometric folding/unfolding motion of an origami cell was simulated, as illustrated in Fig. S2B and C. In this simulation, the rigid sheets were formed of acrylic plate material, and the thickness was set to 1 mm. The membrane was formed of paper material, and the thickness was set to 0.2 mm. The elastic modulus values for the rigid facets and the soft membrane were 3300 MPa and 0.5 MPa, respectively. The Poisson ratio of both rigid facets and the soft membrane was 0.2. The 3D origami model was first constructed in the Creo Parametric software and then imported into ABAQUS software, where the folding/unfolding motion was simulated. In this motion simulation, one side of the origami facet was set to be fixed, and two identical loading forces and were applied to the two corners of the other facet (refer to Fig S2B). The simulation result demonstrated that the origami cell could generate the folding/unfolding motion around the crease with only slight deformation of the two adjacent facets (refer to Fig. S2C). This result confirmed that the simulation could generate a geometric folding/unfolding motion similar to that of the origami cell.

(2) Stiffness behavior simulation of the metamorphic origami unit

Furthermore, the metamorphic origami unit comprised four rectangular rigid facets and four creases. In addition, two couples of creases were collinear when all four rigid facets were coplanar. To verify the stiffness behavior of the metamorphic origami unit, a simulation was also conducted in the ABAQUS software. The simulations used the same materials and construction methods as described above. Two simulations were conducted to analyze the stiffness of the metamorphic origami unit by changing the folding angle  and the elastic modulus of the facets.

In the stiffness simulation of the metamorphic origami unit with changing folding angles, the elastic modulus of the rigid facets and the soft membrane was set to 3300 MPa and 0.5 MPa, respectively, while the Poisson ratio of both rigid facets and the soft membrane was set to be 0.2. Two edges of the facets of the metamorphic origami cell were fixed, and two identical loading forces and were applied to the two edges of the other two facets opposite to the fixed end. The loading forces should not be applied to the membrane directly because the large deformation generated would lead to simulation failure (refer to Fig. S2D). The direction of these two loading forces was along with the bisector of the folding angle. The stiffness at the point could be calculated by measuring the displacement of the point caused by loading forces and along the direction of these two loading forces using the equation provided below.

(1)

The folding angle was set to vary from 0° to 180°. And the result is presented in Fig. 1C.

In the stiffness simulation of the metamorphic origami with changing elastic modulus, the fixed side and the loading forces were demonstrated in Fig. S2D. The elastic modulus of the four rigid facets was set to vary from 2000 MPa to 4000 MPa, and the Poisson ratio was set to 0.2. The elastic modulus and Poisson ratio of the soft membrane were set to 0.5 MPa and 0.2, respectively. The folding angle was . The stiffness at the point could then be calculated by measuring the displacement of the point caused by loading forces and , and the result is presented in Fig. 1D.

Based on the stiffness analysis presented above, the stiffness of this metamorphic origami unit along the direction of loading force can be calculated as

. (2)

(3) Metamorphic motion simulation of the inflatable metamorphic origami unit

The inflatable metamorphic origami unit’s metamorphic behavior was simulated using the following procedures. First, the 3D model of the inflatable metamorphic origami unit was created in the Creo Parametric software and imported into the ABAQUS software. There are eight rigid facets and one soft chamber in the inflatable metamorphic. The rigid facets and soft chamber density were set to 1800 kg/m3 and 970 kg/m3, respectively. The rigid facets and soft chamber elastic moduli were set to 250000 MPa and 900 MPa, respectively. The Poisson ratios of rigid facets and soft chamber were set to 0.3 and 0.45, respectively. The rigid facet's section property was set to solid, while the soft chamber's section property was set to the membrane. The soft chamber mesh element type was M3D4R. Third, the dynamic explicit step was established, as well as the boundary conditions for the inflatable metamorphic origami unit. The gas input side of the inflatable metamorphic origami unit was fixed, and pressure was applied to the soft chamber's inner surface. The pressure was set at 15 Kpa. The simulation result is depicted in Movie S2.

Note S3: Stiffness analysis of the inflatable metamorphic origami unit

Fig. 2A shows an inflatable metamorphic origami unit that is a combination of two four-facet metamorphic origami units in the upper and lower directions. We created an equivalent pin-jointed truss frame model based on the method proposed in [24] to analyze the stiffness behavior of this inflatable metamorphic origami unit. The creases in this model were represented by elastic truss bars, the vertices were represented by frictionless pin-joints, and the virtual bars were added along the facet diagonal lines to approximate the bending of the facets. In this paper, the virtual bar is represented as a virtual crease. The stretching and bending of the facets generated the deformations of this inflatable metamorphic origami unit, which can be represented by the displacements of all vertices. Therefore, the stiffness of the origami unit can be analyzed using the stiffness matrix.

In this analysis, the geometric model was first established to uniquely represent this inflatable metamorphic origami unit using four parameters, i.e., two lengths of the creases and , and the upper and lower folding angles ( and ) between the upper and lower four-facet metamorphic origami units’ facets and the *x-y* plane, as shown in Fig. S3A. Therefore, all vertices in the coordinate system can be represented by the following relationships:

, (3)

where and are the heights of the upper and lower four-facet metamorphic origami units; is the length of the whole inflatable metamorphic origami unit along the *x* direction; and is the width of this unit along the *y* direction.

The stiffness matrices for describing the stretching and bending of this unit were established. The stretching of the whole unit can be represented by the stretching of the creases. And the stretching of each crease is represented by the displacements of the two vertices on the crease. There are 12 vertices () and 28 actual and virtual creases () in this unit, as shown in Fig. S3B. A compatibility matrix can be established to express the displacements of the two vertices for each crease, i.e., , where is the displacement vector of each vertex, and is the crease stretching vector. Thus, (contains the information in *x*, *y*, and *z* directions); and . Since a crease is determined by two vertices and , the stretch of the -th crease can be expressed as

. (4)

And can be calculated as

. (5)

The information of each crease can be obtained using only two vertices, however, to establish a matrix containing all vertices and creases, the -th crease expressed in matrix is . Then, the compatibility matrix contains all vertices and creases is a matrix:

. (6)

The stretching stiffness matrix should take into account the stretching stiffness of the creases and can be obtained as

, (7)

where is the axial stretching stiffness of the creases’ per unit length, and is the length of the -th crease. And is a matrix describing the relationships between 12 vertices.

Furthermore, the bending of the origami unit was represented by changes in the dihedral angles at the actual creases and the facets (virtual creases), as shown in Fig. S3C and Fig. S3D. The dihedral angles are determined by three vectors (Fig. S3E), and their geometric relationships are as follows:

. (8)

There are 20 effective dihedral angles () in this unit; among them, 12 dihedral angles describe the bending at the actual creases (), and 8 dihedral angles express the bending at the facets (). Bending, like stretching, is represented by changes in the corresponding vertices. Thus, the equation is established as , and for the -th dihedral angle, there exists the following equation:

. (9)

The matrix used to describe the changes of dihedral angle also needs to contain the relationships of each dihedral angle corresponding to all vertices. Thus, is established as a matrix, i.e., . Then, the stiffness matrix for the bending should consider the torsional stiffness of the actual creases () and the facets (), and can be obtained a matrix as

. (10)

Finally, the total stiffness matrix can be obtained as .

The eigen stiffness can be calculated using eigen analysis and the total stiffness matrix. The structures with the lowest eigenvalues have the lowest stiffness along the corresponding eigenvectors (except the rigid body motions). Based on this feature of the stiffness matrix, the parameters of the inflatable metamorphic origami unit were set as , , and the relationships between , , and , i.e., and . The eigen stiffness of the stiffness matrices with respect to different upper folding angles () is then shown in Fig. 2B. It can be seen that when , the unit has maximum stiffness. In this configuration, the origami unit is inflated to its maximum volume. And when and , the stiffness of the unit approximates to 0. The unit is in metamorphic positions in these two configurations, and the unit can be rotated around the contiguous creases; thus, the stiffness is almost zero. This method is applicable to other inflatable metamorphic origami.

Note S4: Fabrication of the radial deployable metamorphic origami

The deployable metamorphic origami proposed in the present study was fabricated using a convenient and low-cost method. The developed deployable metamorphic origami comprised three main parts, namely, a soft pneumatic chamber, rigid facets, and a rigid mounting base. The soft pneumatic chamber was fabricated using the flexible polyethylene (PE) lay flat tube. The rigid carbon fiber sheets were used to fabricate the rigid facets of the radial deployable metamorphic origami, the circumferential deployable metamorphic origami, and the multi-fingered deployable metamorphic origami grasper to ensure the stiffness of the metamorphic origami. Because the leaf-shaped deployable metamorphic origami grasper is designed to envelope grasp the objects, the contact area between the grasper and the object is larger to hold the heavy objects. To achieve the adaptive grasping for different size irregular-shaped objects, the rigid facets of the leaf-shaped deployable metamorphic origami grasper were made from elastic polyvinyl chloride (PVC) sheets with higher elasticity than carbon sheets. On the mounting base, the metamorphic origami branches were installed. The key steps in the fabrication process were as follows:

*Step 1*: *Facet attachment* The facets were attached to the outer surface of the flexible PE tube membrane (thickness 0.1 mm) using double-sided sticky tapes. The limited blocks were employed to specify the gap between the adjacent facets (Fig. S4A), which was calculated using the following equation:

(18)

where, denotes the thickness of the rigid facet, denotes the thickness of the double-sided sticky tape, and denotes the thickness of the PE tube membrane.

*Step 2*: *Tube membrane sealing* One end of each flexible PE tube membrane was sealed using the heat-sealing method (refer to Fig. S4B). After sealing, a semi-closed chamber was obtained.

*Step 3*: *Wrinkle generation* The wrinkled region between adjacent facets was generated by squeezing the membrane. In order to obtain stretchable creases, this wrinkled structure was required as it would generate both folding/unfolding motion and stretching motion. In order to obtain a rigid crease, a tensile tape was used for gluing together adjacent facets to limit the stretching motion such that only the folding/unfolding motion would be generated (Fig. S4C).

*Step 4*: *Branch installation* The non-sealed end of the origami branch was installed on the mounting interface of the base using double-sided sticky tape (Fig. S4D). The tightness of the branch was improved by sealing the connection position of the PE tube membrane and the mounting base using a sealant (Fig. S4E).

The radial deployable metamorphic origami was designed to support a deployable planar solar array in space engineering applications. This metamorphic origami comprised four identical branches and a mounting base. In addition, only rigid creases were present on each branch of the unit as only deployment motion was required to be performed using this prototype.

In the branches of the radial deployable metamorphic origami, the chambers were inflated to a deployed configuration with a rhomboid cross-section to ensure a satisfactory stiffness level for supporting the loads (refer to Fig. S5A). In order to fabricate the branches of the radial deployable metamorphic origami, the flexible PE lay flat tube was first used for fabricating the soft pneumatic chamber, following which the rectangular carbon fiber sheets adhered to the PE lay flat tube using double-sided sticky tapes (Fig. S5B). This prototype contained one short module and five long modules (twice as long as the shot module) in each branch. The short module was used for the central alignment of the folded branch to the base. Moreover, each module comprised four identical rectangular carbon fiber sheets (Fig. S5A). The specified sizes of the gaps between adjacent facets of the adjacent modules must consider the thickness of the carbon fiber sheets, the thickness of double-sided sticky tapes, and the thickness of the PE lay flat tube to ensure a completely-folded configuration of the origami. The gap size specifications and the corresponding fabrication approach are illustrated in Fig. S4A. After facets attachment, one end of the PE lay flat tube attached with long modules was sealed by a heat sealer to form a semi-closed chamber (Fig. S5C). Finally, the double-sided sticky tape was used to attach the non-sealed end of the branch to the mounting interface of the base (Fig. S5D). The open end of the PE lay flat tube was glued to the mounting base using the sealant (Fig. S5E).

The solar array was imitated by attaching the PVC sheets to a large membrane paper and was mounted on the four branches of the radial deployable metamorphic origami using soft cables (Fig. S5F and Fig. S5G).

Note S5: Fabrication of the circumferential deployable metamorphic origami

The circumferential deployable metamorphic origami was designed to support a deployable curved-surface antenna in space engineering applications. This metamorphic origami comprised four identical branches and a mounting base. Both rigid creases and stretchable creases were included in this origami as both deployment motion and bending motion were required to be performed using this prototype. The branch of the circumferential metamorphic origami had a triangular cross-section in the completely-deployed configuration (Fig. S6A). The prototype contained five modules with different lengths for each branch, and these modules had to be folded circumferentially around the mounting base (Fig. 4A). In addition, two pairs of identical rectangular facets were present in each module. A pair of narrow facets was attached to one lateral face of the triangular prism membrane chamber, and a pair of wide facets was attached to the other two lateral faces (Fig. S6A).

All the facets of the origami were formed of carbon fiber sheets that were adhered to the PE lay flat tube using double-sided sticky tapes (Fig. S6B). Similar to the radial deployable metamorphic origami, the specified sizes of the gaps between facets of the adjacent modules must consider the thickness of the carbon fiber sheets, the thickness of the double-sided sticky tapes, and the thickness of the PE lay flat tube to ensure a completely-folded configuration of the origami and to generate wrinkles for stretchable creases. The gap specifications and the fabrication approach are presented in Fig. S4A. Afterward, the end of the PE lay flat tube attached to the facets of the last module was sealed by the heat sealer (Fig. S6C). Then, the wrinkles between the facets in adjacent modules were generated by squeezing the membrane at the gap (Fig. S6D). A tensile tape was then used for gluing the narrow facets in one lateral face of the triangular prism such that the creases in this face with wrinkled structures formed rigid creases while the stretchable creases were generated between the adjacent wide facets (Fig. S6E). Finally, the other end of the PE lay flat tube was mounted on the mounting interface of the base (Fig. S6F). The tightness of this chamber was improved by sealing the connection position of the PE lay flat tube and the mounting base using a sealant (Fig. S6G). The circumferential metamorphic origami was obtained after four identical branches were fabricated and installed on the mounting base (Fig. S6H). The deployable curved-surface antenna supported by the above metamorphic origami comprised four identical parts, which were, respectively, mounted on the four branches of the circumferential metamorphic origami (Fig. S6I). The physical prototype of this metamorphic origami was fabricated (Fig. 4C), and the deployment process of this prototype was evaluated (Fig. 4D). Furthermore, to reduce the influence of gravity in deployment performance of the circumferential metamorphic origami, a gravity compensation system using fishing lines was used to simulate weightlessness in space.

Note S6: Fabrication of the multi-fingered deployable metamorphic origami grasper

The multi-fingered deployable metamorphic origami grasper was designed for grasping large-sized objects. This metamorphic origami comprised three identical branches and a mounting base. Both rigid creases and stretchable creases were included in this origami as both deployment motion and bending motion were required to be performed using this prototype.

In the branches of the multi-fingered deployable metamorphic origami grasper, the deployed configuration of the soft chamber made of PE lay flat tube was designed with a rectangular cross-section. The branch could generate both deployment and bending motions sequentially. Each module was formed of two identical wide rectangular carbon fiber sheets and four identical narrow rectangular carbon fiber sheets (Fig. S7A). First, the carbon fiber sheets as facets of the metamorphic origami were attached to the PE lay flat tube using double-sided sticky tapes (Fig. S7B). This prototype contained five identical modules for each branch. The width of the wide carbon fiber sheets was twice that of the narrow carbon fiber sheets. Four narrow carbon fiber sheets were attached opposite to a pair of lateral faces of the rectangular prism chamber, and two wide carbon fiber sheets were attached opposite to another pair of lateral faces of the rectangular chamber. The specified sizes of the gaps between adjacent facets of the adjacent modules must consider the thickness of the carbon fiber sheets, the thickness of the double-sided sticky tapes, and the thickness of the PE lay flat tube to ensure the completely-folded configuration of the origami and to produce wrinkles forming stretchable creases. The gap fabrication approach was similar to the one described for the previous prototypes. Afterward, the end of the PE lay flat tube was sealed by the heat sealer (Fig. S7C). Then, the wrinkles between adjacent wide carbon fiber sheets in the adjacent modules were generated by squeezing the membrane at the gap (Fig. S7D). On the side where the wide carbon fiber sheets were attached, a tensile tape was used for gluing the adjacent rigid facets to form the rigid crease. On the other side where wide carbon fiber sheets were attached; the soft membrane was squeezed to form the stretchable crease (Fig. S7E). The rigid crease ensured the geometric folding/unfolding motion. The wrinkled structure of the stretchable crease could generate both folding/unfolding motion and stretching motion. Finally, a double-sided sticky tape was used for attaching the non-sealed end of the branch to the mounting interface of the base, and the connection position of the tube membrane and the mounting base was sealed using a sealant (Fig. S7F and Fig. S7G). The prototype of the multi-fingered deployed metamorphic origami grasper was obtained after the mounting of these three branches (Fig. S7H).

Note S7: Fabrication of the leaf-shaped deployable metamorphic origami grasper

The leaf-shaped deployable metamorphic origami grasper was designed for grasping large-sized heavy objects. This metamorphic origami comprised three identical leaf-shaped branches and a mounting base. Both rigid creases and stretchable creases were included in this origami as both deployment motion and envelope motion were required to be performed using this prototype. The leaf-shaped deployable metamorphic origami branch contained two kinds of creases: rigid creases and curved stretchable creases. The grasping of heavy objects could be realized because of the wide leaf-shaped branches conferring the envelope grasping mode.

In the leaf-shaped branch of the metamorphic origami grasper, the wide flexible PE lay flat tube was used for fabricating the soft pneumatic chamber. The green PVC sheets of different sizes were attached to one side of the PE lay flat tube using double-sided sticky tapes and were used as facets of the metamorphic origami (Fig. S8A). In comparison to carbon fiber sheets, PVC sheets have greater flexibility for adaptive grasping. When these PVC sheets were attached to the PE lay flat tube, the whole pattern appeared similar to a leaf. Similar to the previous prototype, the specified sizes of the gaps between the adjacent facets must consider the thickness of the PVC sheets, the thickness of the double-sided sticky tapes, and the thickness of the PE lay to ensure the completely-folded configuration of the origami. The heat sealer was used to seal the edge of the leaf-shaped branch and unnecessary leftovers were removed (Fig. S8B). In order to prevent the leaf-shaped deployable metamorphic origami grasper from bulging during inflation along the longitudinal crease, the central regions of the PE lay flat tube of the leaf-shaped branch were also sealed (indicated by the segmented red line in Fig. S8B). The sealing lines were realized using the heat insulation tape outside the chamber during sealing, (indicated by blue rectangles in Fig. S8B). The wrinkled between adjacent PVC sheets were generated by squeezing the membrane at the gap, as described in the previous prototype (Fig. S8C). On one side of the PE lay flat tube where the green PVC sheets were attached, the tensile tape was used for attaching the adjacent rigid facets to form the rigid crease, and these rigid creases contained both lateral creases and longitudinal creases (Fig. S8D). On the opposite side, without the PVC sheets, the PE membrane was squeezed to form the curved stretchable crease. The wrinkled structures of the curved stretchable crease could generate both folding/unfolding motion and stretching motion for the side without PVC sheets. Consequently, the three leaf-shaped deployable metamorphic origami branches could generate a bending motion for grasping objects. Finally, a double-sided sticky tape was used to attach the non-sealed end of the leaf-shaped branch to the mounting interface of the base (Fig. S8E), and the connection position of the tube membrane and the mounting base was sealed using a sealant (Fig. S8F). The prototype of the leaf-shaped deployable metamorphic origami grasper was fabricated after these three leaf-shaped branches were mounted (Fig. S8G).

Note S8: The self-folding ability design of the metamorphic origami

Although the inflatable metamorphic origami can show a sequential motion process when the soft chamber is inflated, the origami can not be folded into the initial compact folded configuration without the use of an outside actuation force. This problem can be solved by using elastic steel wires. Based on the elastic force of the elastic steel wires, the four metamorphic origami can achieve the self-fold. To achieve the self-folding ability, the arrangement of the elastic steel wires on the four different metamorphic origami is as follows:

(1) For the radial deployable metamorphic origami, two elastic steel wires can be attached to the two sides of each branch, as shown in Fig. S9A.

(2) For the circumferential deployable metamorphic origami, two elastic steel wires can be attached to the two top-side creases for each branch, as shown in Fig. S9B.

(3) For the multi-fingered deployable metamorphic origami grasper, two elastic steel wires were attached to the two top-side rigid creases for each branch, as shown in Fig. S9C.

(4) For the leaf-shaped deployable metamorphic origami grasper, an elastic steel wire can be attached to the middle crease of each leaf-shaped branch, as shown in Fig. S9D.


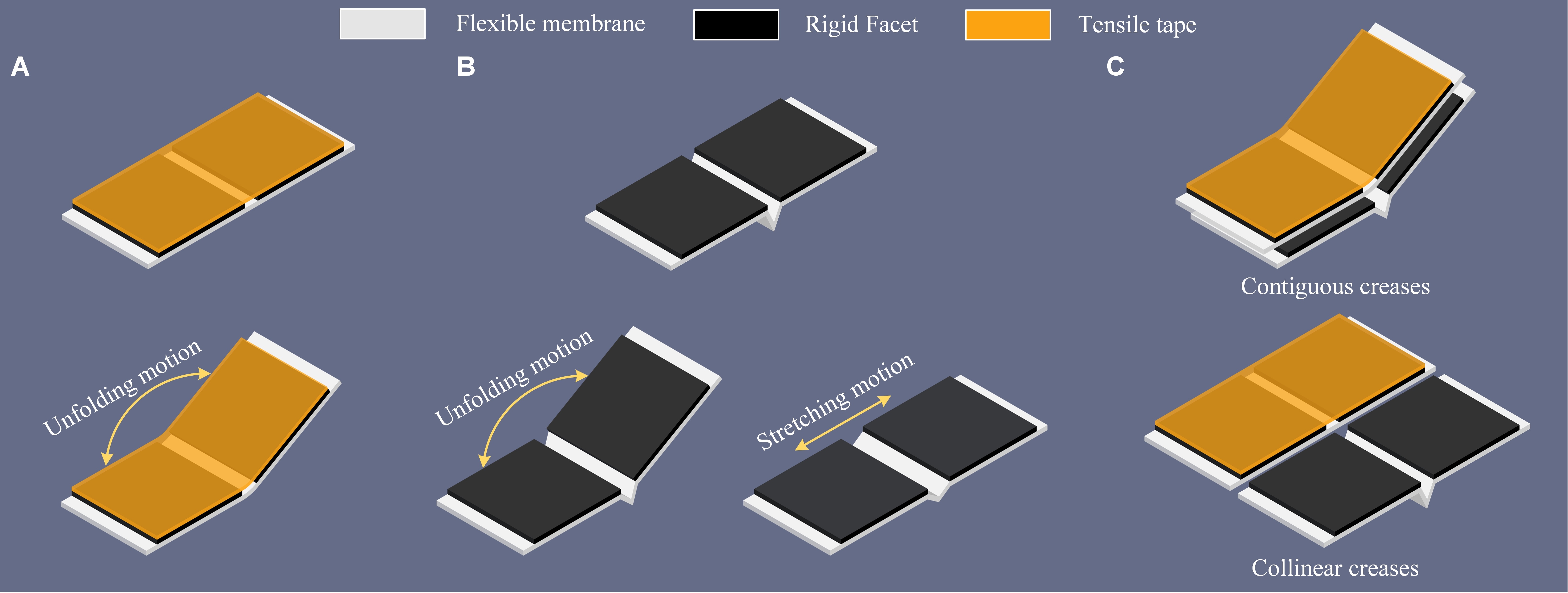


**Fig. S1. Design of the origami creases.** (A) Rigid crease. (B) Stretchable crease. (C) Contiguous creases and collinear creases.


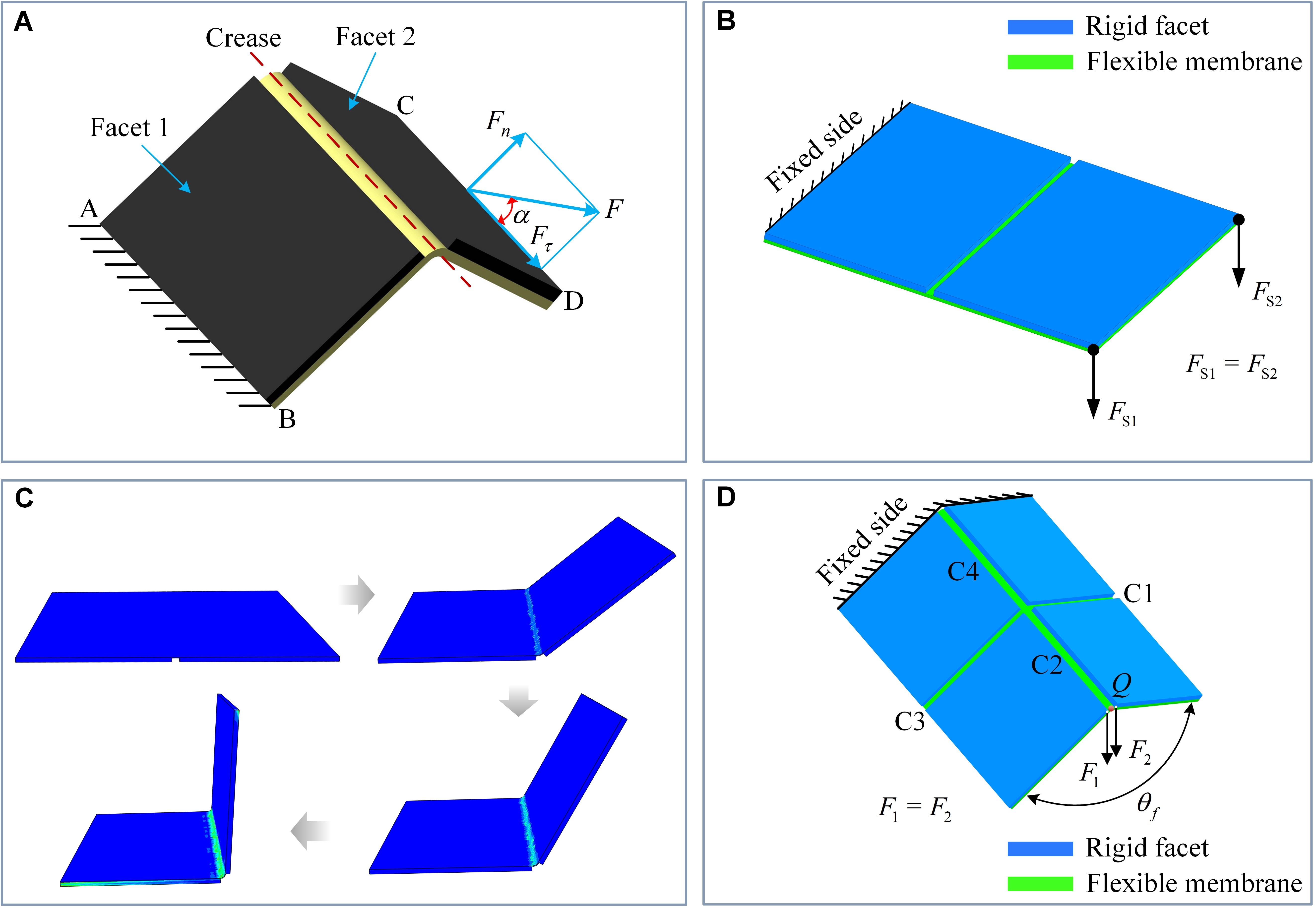


Fig**.** S2**. Simulation of the origami cell and metamorphic origami unit.** (A) Origami cell model with single-crease. (B) Setup for the origami cell simulation. (C) Simulation of the folding/unfolding motion of the origami cell. (D) Simulation model for the metamorphic origami unit.


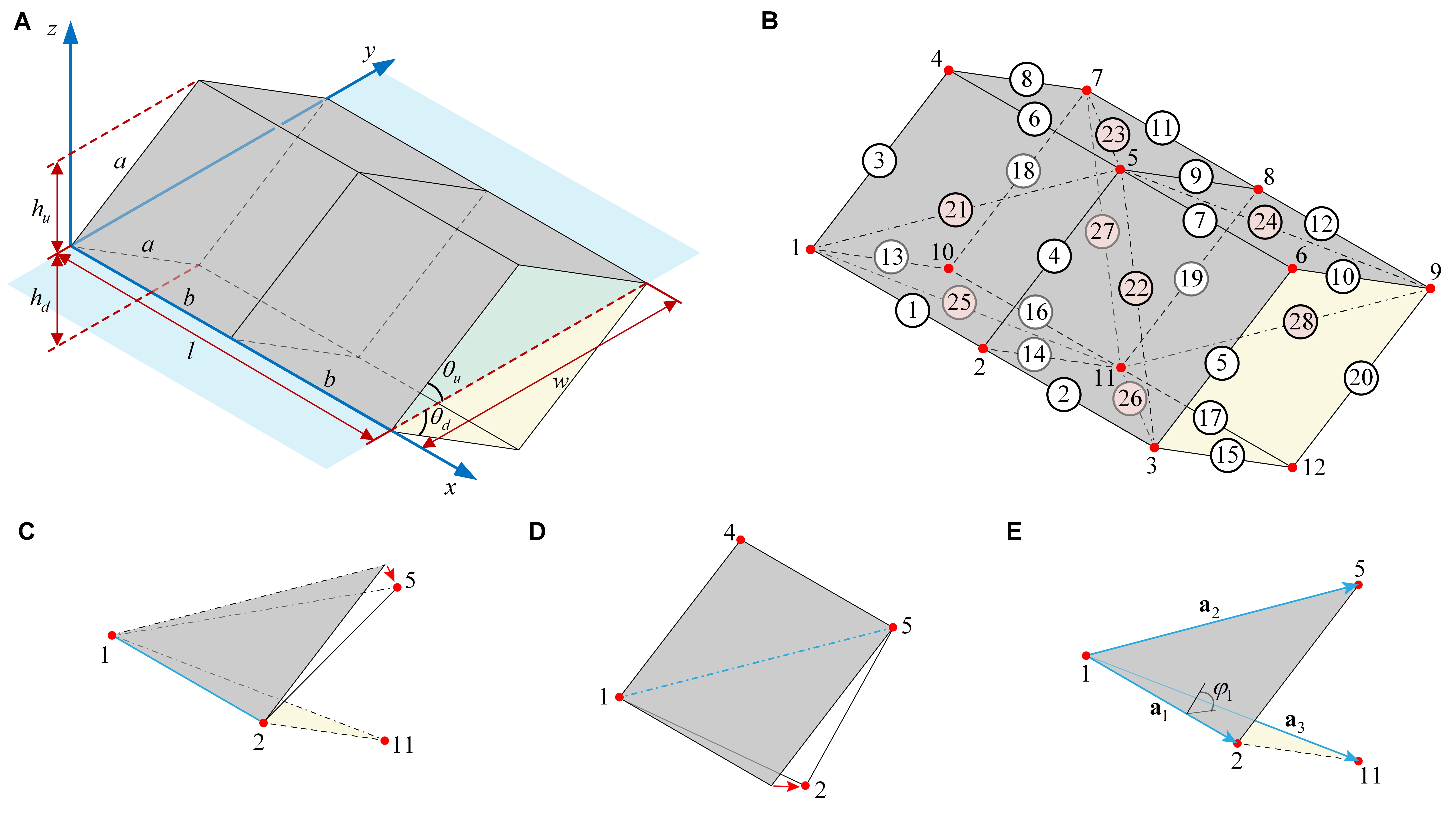


**Fig**. **S3. Geometric model of the inflatable metamorphic origami unit.** (A) Parameters of the inflatable metamorphic origami unit. (B) Pin-jointed truss frame model. (C) Bending motion approximation of the crease in the unit. (D) Bending motion approximation of the facet in the unit. (E) Definition of a dihedral angle between two adjacent facets.


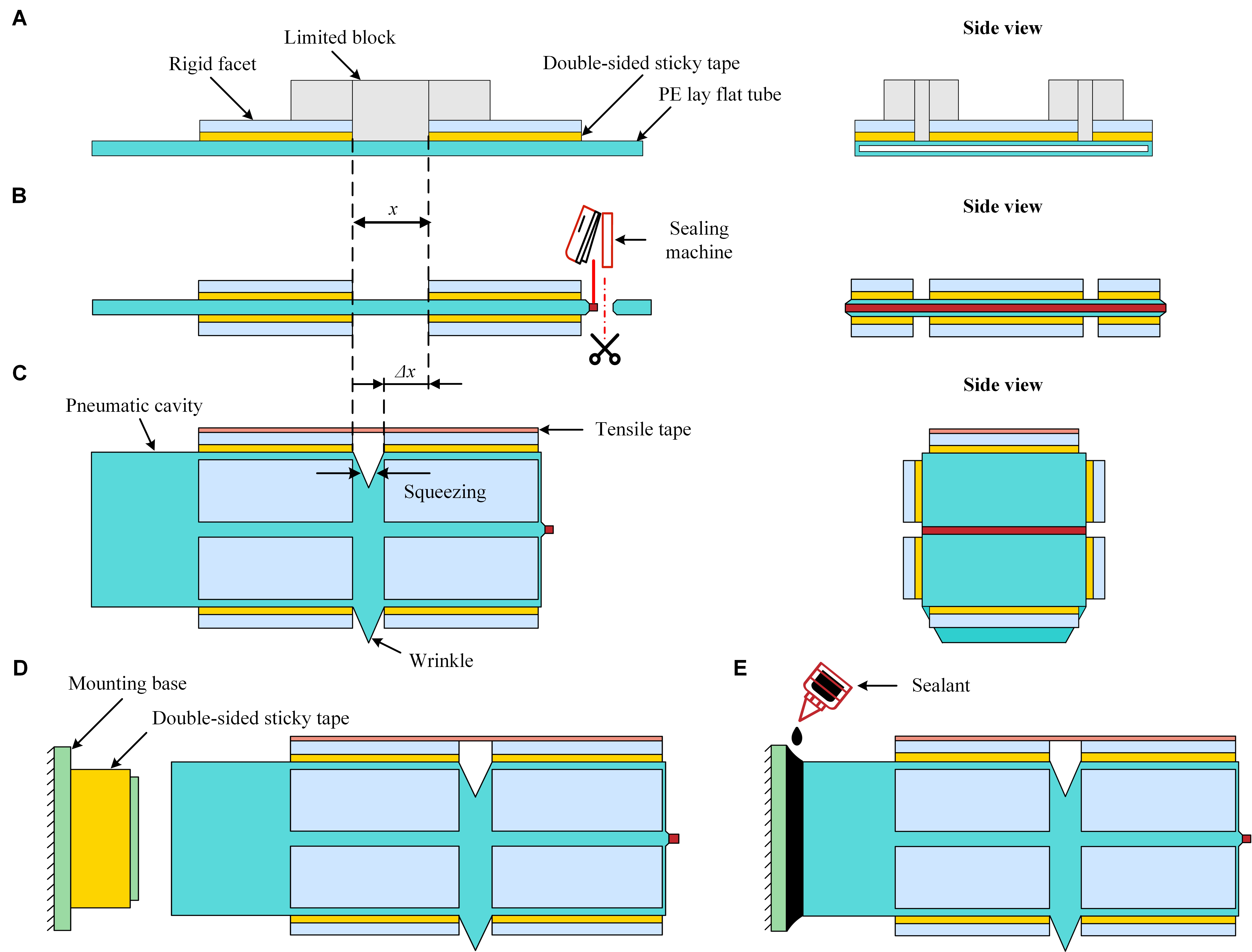


**Fig**. **S4. The fabrication process of the deployable metamorphic origami prototype.** (A) The facets of the developed metamorphic origami were attached to the tube membrane. (B) The tube membrane was sealed to form a semi-enclosed pneumatic chamber. (C) Wrinkles were generated between adjacent facets. (D) Branch was installed on the mounting base. (E) Tightness improvement was performed.


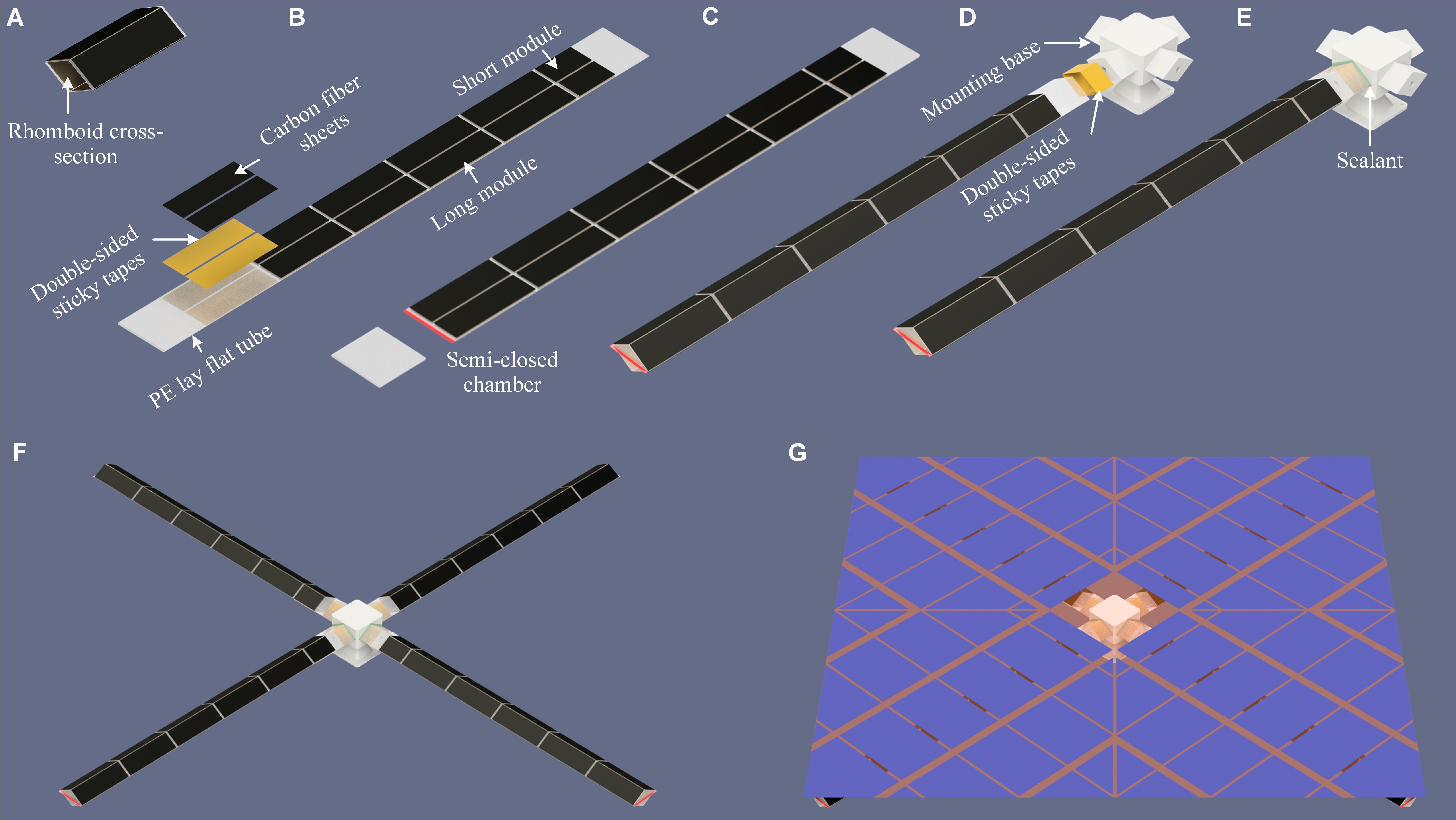


**Fig**. **S5. The fabrication process of the radial deployable metamorphic origami.** (A) The deployed module with rhomboid cross-section. (B) The facets of the metamorphic origami were attached to the PE lay flat tube membrane. (C) The tube membrane was sealed to form a semi-enclosed pneumatic chamber. (D) Branch was installed on the mounting base. (E) Tightness improvement was performed. (F) Four branches were installed on the mounting base to form the radial metamorphic origami. (G) The deployable solar array was mounted on the four branches.


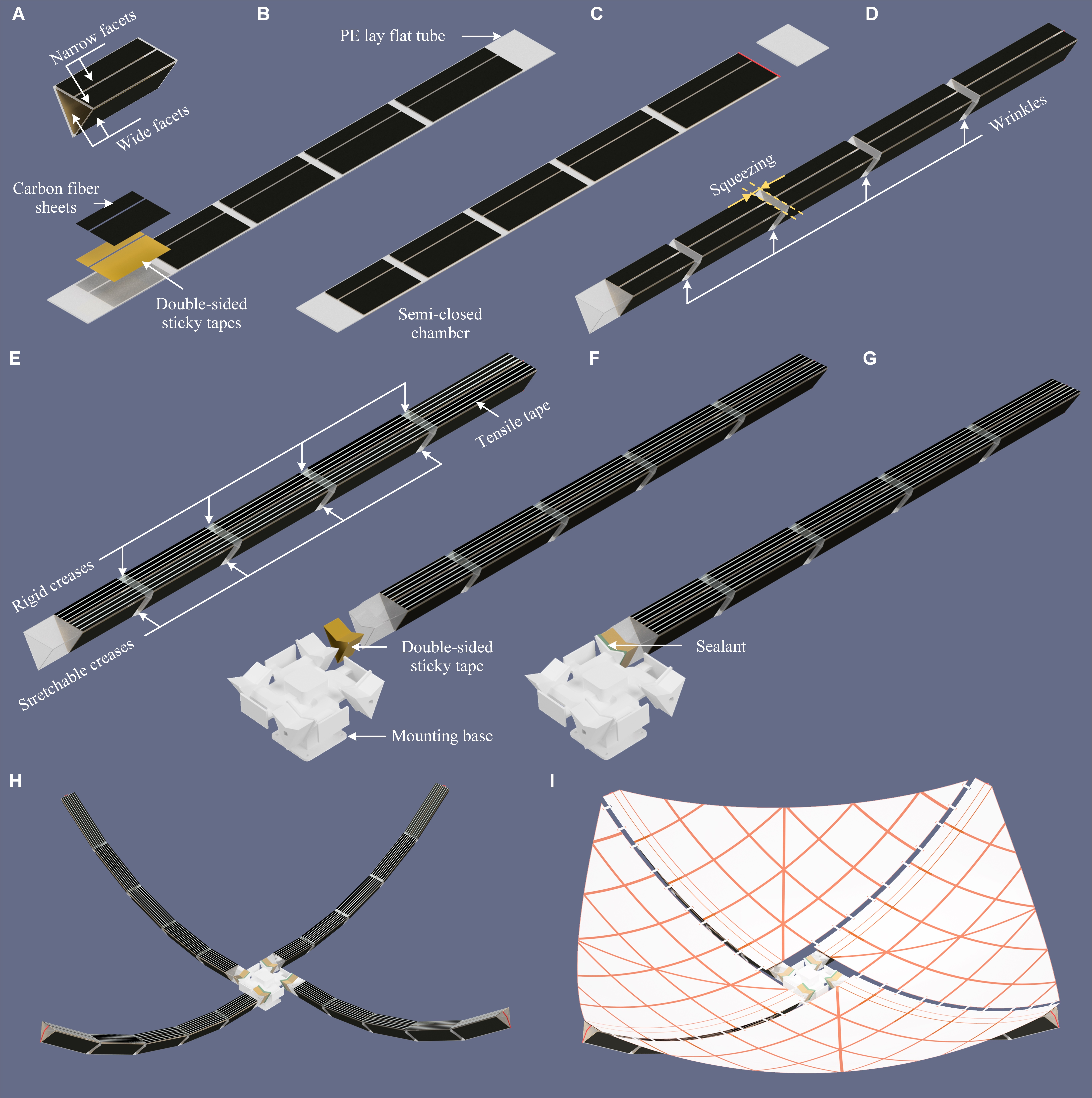


Fig. S6. The fabrication process of the circumferential deployable metamorphic origami. (A) The deployed module with a triangular cross-section. (B) The carbon fiber sheets were attached to the tube membrane. (C) The tube membrane was sealed to form a semi-enclosed pneumatic chamber. (D) Wrinkles were generated between adjacent facets through squeezing. (E) A tensile tape was used for gluing one lateral face to the generated rigid creases. (F) The branch was installed on the mounting base using double-sided sticky tape. (G) Tightness improvement was performed. (H) The circumferential deployable metamorphic origami with its four branches. (I) The deployable curved-surface antenna was mounted on the metamorphic origami.


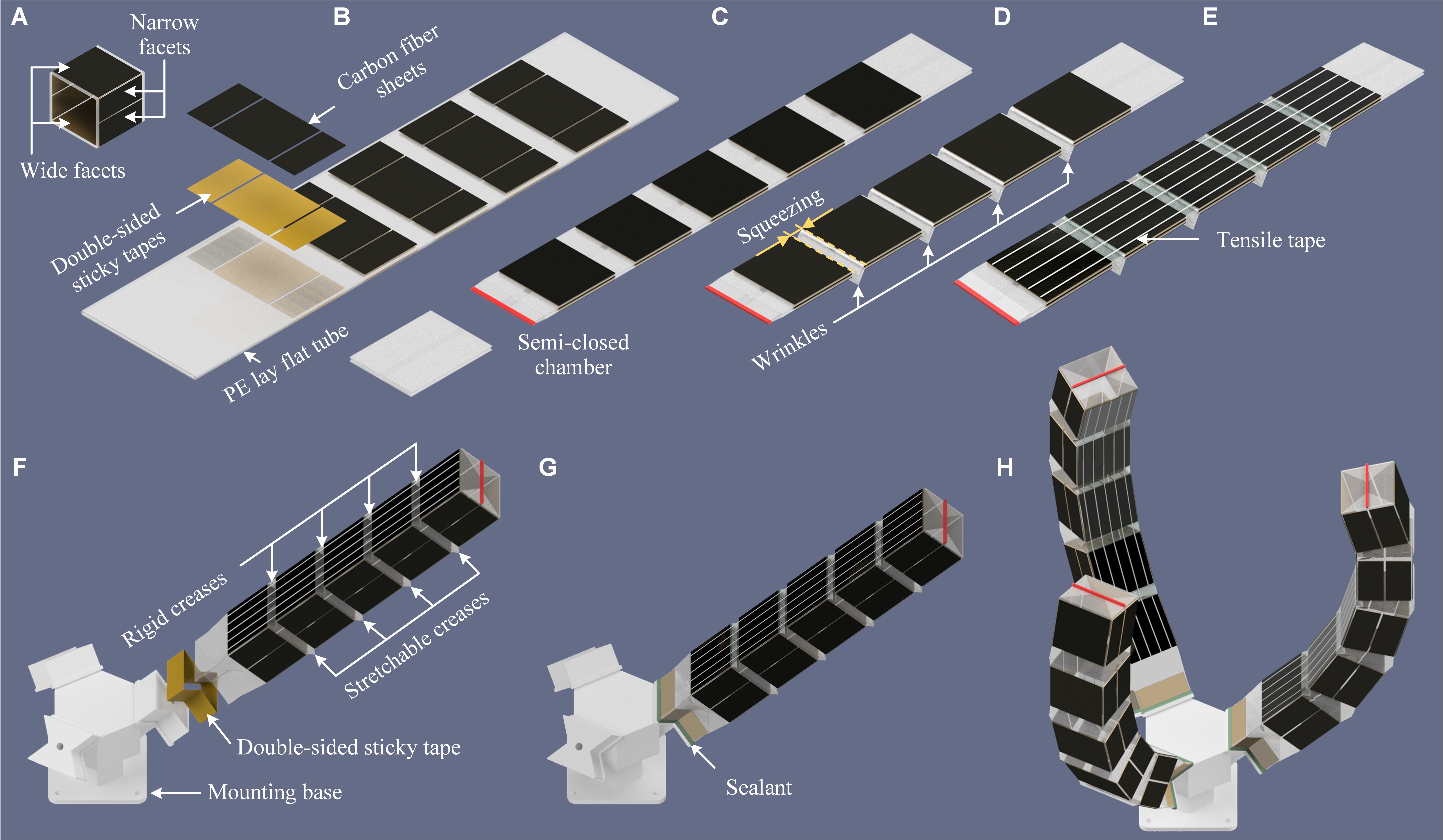


Fig. S7. The fabrication process of the multi-fingered deployable metamorphic origami grasper. (A) The deployed module with a rectangular cross-section. (B) The carbon fiber sheets were attached to the tube membrane. (C) The tube membrane was sealed to form a semi-enclosed pneumatic chamber. (D) Wrinkles were generated between the adjacent facets through squeezing. (E) A tensile tape was employed to glue the one lateral face with the wide rigid facets. (F) The branch was installed on the mounting base using double-sided sticky tape. (G) Tightness improvement was performed. (H) The multi-fingered deployable metamorphic origami grasper.


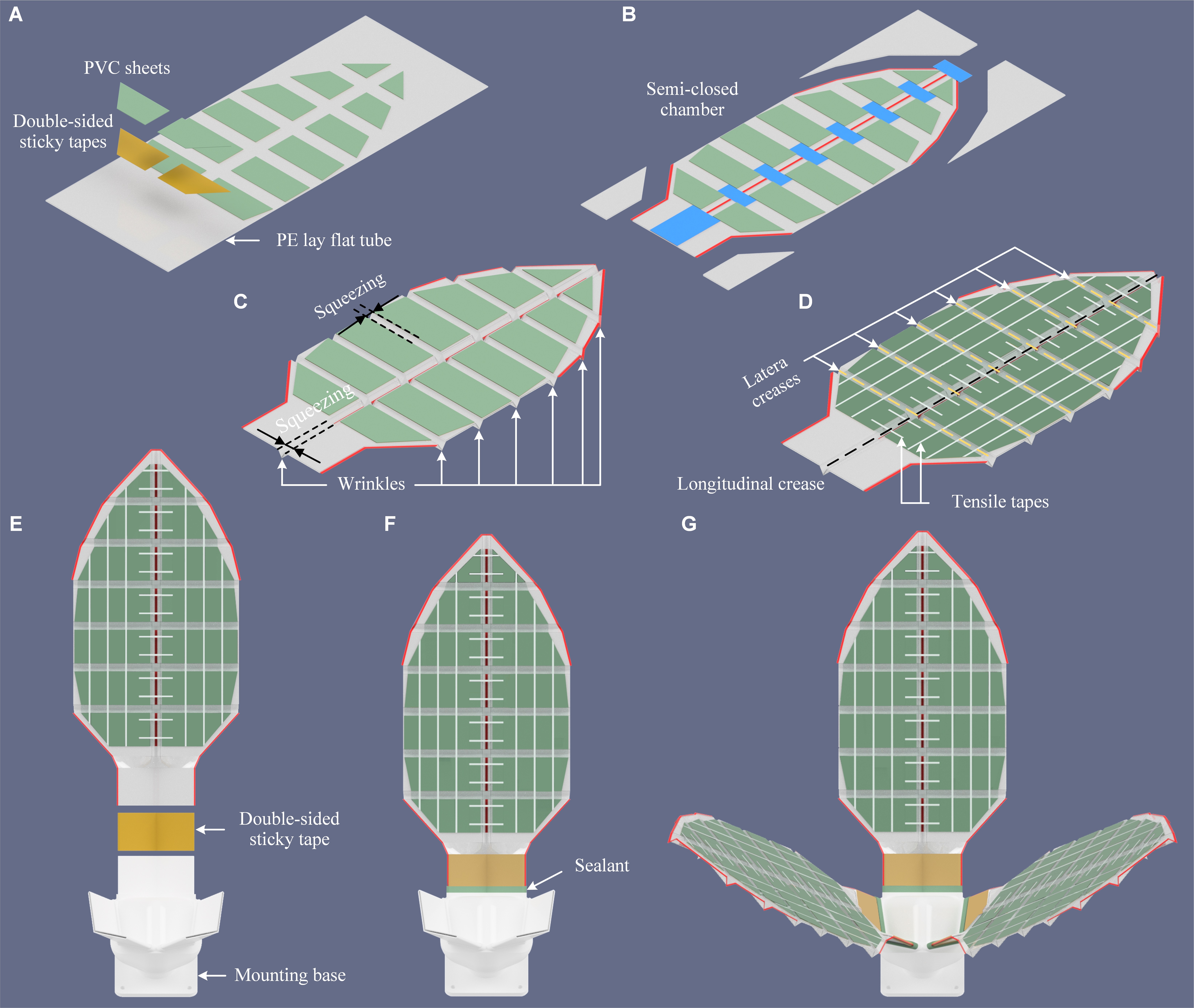


Fig. S8. The fabrication process of the leaf-shaped deployable metamorphic origami grasper. (A) The PVC sheets were attached to the tube membrane. (B) The tube membrane was sealed to form a semi-enclosed pneumatic chamber. (C) Two types of wrinkles were generated through squeezing. (D) Tensile tapes were employed to attach the adjacent rigid facets on the upper side of the leaf-shaped branch. (E) The leaf-shaped branch was installed on the mounting base using double-sided sticky tape. (F) Tightness improvement was performed. (G) The leaf-shaped deployable metamorphic origami grasper with three branches was completely fabricated.


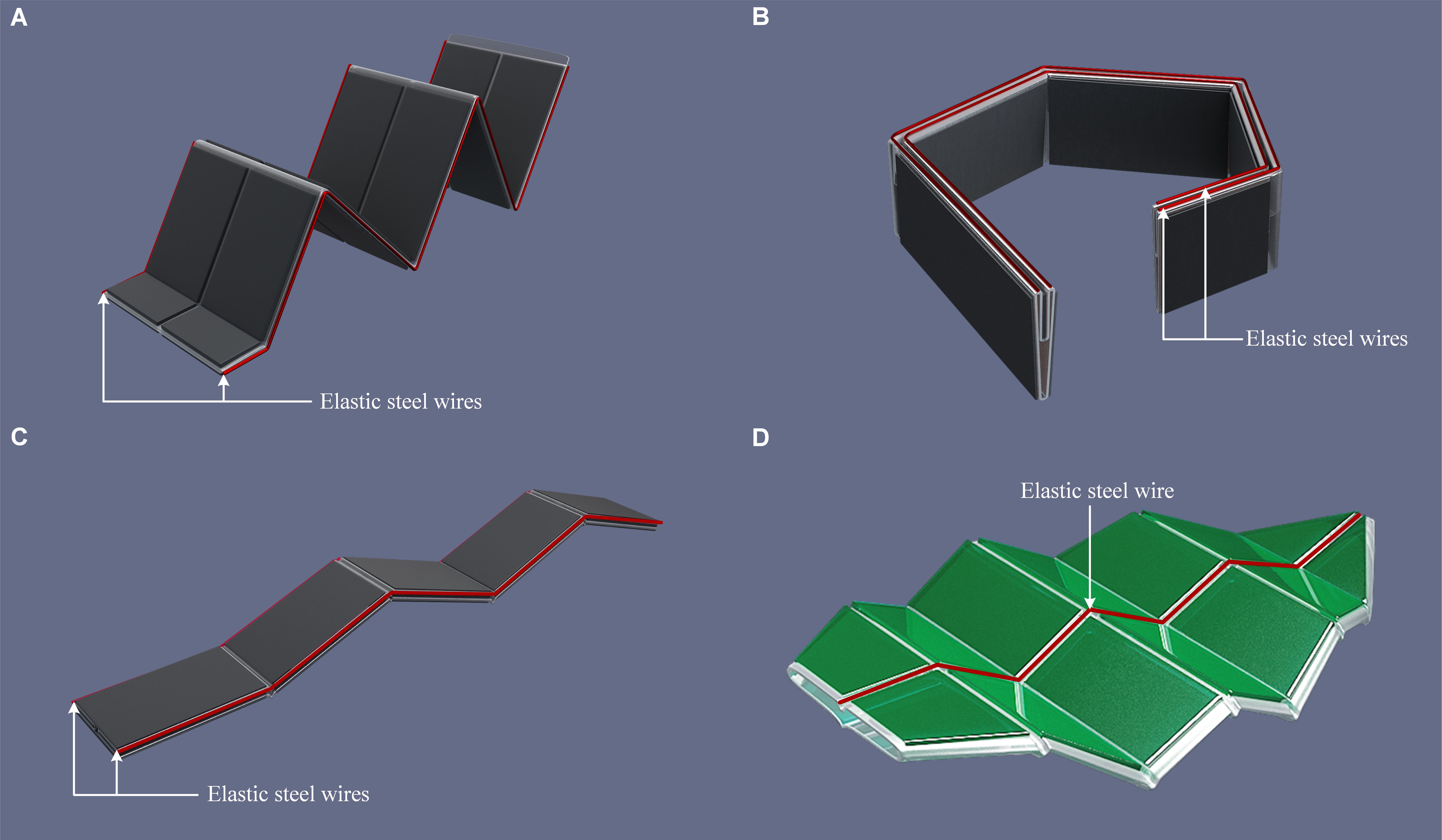


Fig. S9. The arrangement of the elastic steel wires for self-folding. (A) The elastic steel wires on radial deployable metamorphic origami. (B) The elastic steel wires on circumferential deployable metamorphic origami. (C) The elastic steel wires on multi-fingered deployable metamorphic origami grasper. (D) The elastic steel wire on leaf-shaped deployable metamorphic origami grasper.
